# The budding yeast protein Chl1p is required for delaying progression through G1/S phase after DNA damage

**DOI:** 10.1186/s13008-021-00072-x

**Published:** 2021-09-08

**Authors:** Muhseena N. Katheeja, Shankar Prasad Das, Suparna Laha

**Affiliations:** 1 Cell Biology and Molecular Genetics Division, Yenepoya Research Centre, Yenepoya Medical College, Yenepoya (Deemed To Be University), University Road, 3rd floor, Academic block, Deralakatte, Mangalore, 575018 India; 2 Department of Biochemistry, Bose Institute, P1/12 CIT Scheme VII M, 700 054 Kolkata, India

**Keywords:** Yeast, Chl1p, Checkpoint, Bud-emergence, DNA damage, G1/S phase, DNA repair

## Abstract

**Background:**

The budding yeast protein Chl1p is a nuclear protein required for sister-chromatid cohesion, transcriptional silencing, rDNA recombination, ageing and plays an instrumental role in chromatin remodeling. This helicase is known to preserve genome integrity and spindle length in S-phase. Here we show additional roles of Chl1p at G1/S phase of the cell cycle following DNA damage.

**Results:**

G1 arrested cells when exposed to DNA damage are more sensitive and show bud emergence with faster kinetics in chl1 mutants compared to wild-type cells. Also, more damage to DNA is observed in *chl1* cells. The viability falls synergistically in *rad24chl1* cells. The regulation of Chl1p on budding kinetics in G1 phase falls in line with Rad9p/Chk1p and shows a synergistic effect with Rad24p/Rad53p. *rad9chl1* and *chk1chl1* shows similar bud emergence as the single mutants *chl1*, *rad9* and *chk1.* Whereas *rad24chl1* and *rad53chl1* shows faster bud emergence compared to the single mutants *rad24*, *rad53* and *chl1*. In presence of MMS induced damage, synergistic with Rad24p indicates Chl1p’s role as a checkpoint at G1/S acting parallel to damage checkpoint pathway. The faster movement of DNA content through G1/S phase and difference in phosphorylation profile of Rad53p in wild type and *chl1* cells confirms the checkpoint defect in *chl1* mutant cells. Further, we have also confirmed that the checkpoint defect functions in parallel to the damage checkpoint pathway of Rad24p.

**Conclusion:**

Chl1p shows Rad53p independent bud emergence and Rad53p dependent checkpoint activity in presence of damage. This confirms its requirement in two different pathways to maintain the G1/S arrest when cells are exposed to damaging agents. The bud emergence kinetics and DNA segregation were similar to wild type when given the same damage in nocodazole treated *chl1* cells which establishes the absence of any role of Chl1p at the G2/M phase. The novelty of this paper lies in revealing the versatile role of Chl1p in checkpoints as well as repair towards regulating G1/S transition. Chl1p thus regulates the G1/S phase by affecting the G1 replication checkpoint pathway and shows an additive effect with Rad24p for Rad53p activation when damaging agents perturb the DNA. Apart from checkpoint activation, it also regulates the budding kinetics as a repair gene.

**Supplementary Information:**

The online version contains supplementary material available at 10.1186/s13008-021-00072-x.

## Background

The helicase Chl1p is a nuclear protein required for sister-chromatid cohesion in mitosis and meiosis [1–3], transcriptional silencing, recombinant DNA (rDNA) recombination, ageing and plays an instrumental role in chromatin remodeling [1, 4–6]. It preserves genome integrity upon DNA damage in S-phase [7]. Chl1p protects cells against DNA damage arising from endogenous or exogenous DNA insults which reveals the requirement of this protein in the repair of DNA damage. The three highly related human homologs of Chl1p are BACH1, hChlR1 and hChlR2. hChlR1 and hChlR2 are expressed only in proliferating human cell lines. Of these, hChlR1 shows in vitro DNA helicase activity and binds to both single- and double-stranded DNA [8, 9]. BACH1(Breast Cancer Associated C terminal Helicase 1) is a member of the DEAH helicase family and binds to the Rad9p homolog BRCA1, contributing towards DNA repair activity [10].

In the yeast *Saccharomyces cerevisiae,* three DNA damage-inducible checkpoints have been identified that operate in G1, S, and G2 phases of the cell cycle [11–16]. Two checkpoints activate prior to S-phase checkpoints in response to DNA damage—one at G1 and the other at G1/S [12, 13] and both of them are Rad9p dependent. At low levels of drug concentrations, DNA damage activates Rad53p only in S-phase and requires the formation of replication forks [17]. When the treatment with MMS is at higher concentrations or for longer periods, DNA damage causes Rad53p activation outside S-phase, leading to G1/S or G2/M arrest [17–19]. Two genes, Mitosis Entry Checkpoint protein 1 (*MEC1/ESR1/SAD3)* and Mitosis Entry Checkpoint protein 2 *(RAD53/MEC2/SPK1/SAD1)* appear important for the performance of all three checkpoints [14, 15, 20, 21]. In case of DNA breaks due to genotoxic agents, the two phosphoinositide 3 kinase-related kinases (PI3KKs), Mec1 and Tel1, the replication factor-C (RFC) like complex consisting of RFC1-like protein Rad24p with four small RFC subunits (Rfc2– Rfc5), the proliferating cell nuclear antigen (PCNA)-like heterotrimeric ring consisting of Rad17, Ddc1 and Mec3 proteins and the MRX complex of proteins, consisting of Mre11, Rad50 and Xrs2 acts as sensors and are recruited at the site of damage to activate the downstream kinases [22–27]. They transmit the signal to the adaptor/mediator molecule, Rad9p, which is activated by phosphorylation in a Mec1/Tel1-dependent fashion. *RAD9* was the first DNA damage checkpoint gene identified in the yeast *Saccharomyces cerevisiae* and was found to play a role in ionizing radiation induced G2/M cell cycle arrest [28–33]. Throughout the cell cycle, it is required for activation of kinase Rad53p in response to DNA double stranded breaks. Another checkpoint kinase, Chk1p, in addition to Rad53p has an apparently minor role in budding yeast during M-phase and G2 phase only [34, 35]. Its activation is also dependent on Rad9p [36]. In addition to this, *RAD9, RAD17, RAD24,* and *MEC3* are involved in G1 and G2 checkpoints [12–14]. Two independent mechanisms exists for the Rad9p activity- the Tudor/BRCA1 C-terminus (BRCT) domains of Rad9p plays the role of Rad53p activation at G1/S phase and the Cyclin Dependent Kinase (CDK) consensus sites of Rad9p activates Rad53p at G2/M [37, 38]. Rad9p homologs 53BP1, MDC1 and BRCA1 also modulates the checkpoint pathways at two phases of the cell cycle. Activation of Rad53p at G1/S depends on the association of Rad9p with the modified chromatin surrounding the double strand breaks. This is mediated by the binding of Tudor/BRCT domain of Rad9p with di-methylated histone H3 and to phosphorylated histone H2A respectively [37]. Any mutation in the pocket fail to execute the G1 checkpoint delay, but the G2/M arrest induced by Nocodazole is well maintained in presence of the same mutations. Furthermore, the binding of Rad9p to histone H2A maintains the G1 checkpoint delay instead of the phosphorylation of H2A, when challenged with xenotoxic agents [14, 37]. Thus, the delay of S-phase following treatment with DNA damaging agents is an actively regulated response that requires functional *RAD9* and *RAD24* genes [12, 13].

In this paper, we have observed the same characteristics in chl1 mutants. Like *rad9*, chl1 mutants also fail to execute the G1 arrest when treated with Methyl Methane Sulphonate (MMS). This study shows that Chl1p is essential for G1/S arrest in response to DNA damage and it acts in line with Rad9p. In presence of a pulse of damage, the *chl1* cells show faster kinetics of bud emergence when compared to the wild type cells indicative of a compromised checkpoint function. To understand the status of checkpoints at G1/S in presence of damage, alpha-factor treated G1 arrested cells were exposed to genotoxic agent MMS. We observed the bulk DNA accumulation along with compromised Rad53p phosphorylation in *chl1* mutant cells at G1/S phase of the cell cycle, which are the hallmark characteristics of checkpoint proteins. The above mentioned observations confirm the early entry into S-phase for *chl1* mutant cells is due to defect in checkpoints compared to wild-type cells. We also observed that apart from the checkpoint defect of Chl1p which is Rad53p dependent, it follows an additional pathway to regulate the bud emergence at G1/S upon DNA damage as the bud emergence of *rad53chl1* is additive to single mutants *rad53* and *chl1*. All these findings confirm the dual role of this protein in controlling the G1 to S transition in the cell cycle on exposure to DNA damage.

## Results

### Chl1p is required for G1/S arrest after DNA damage by MMS

**Fig.1:**
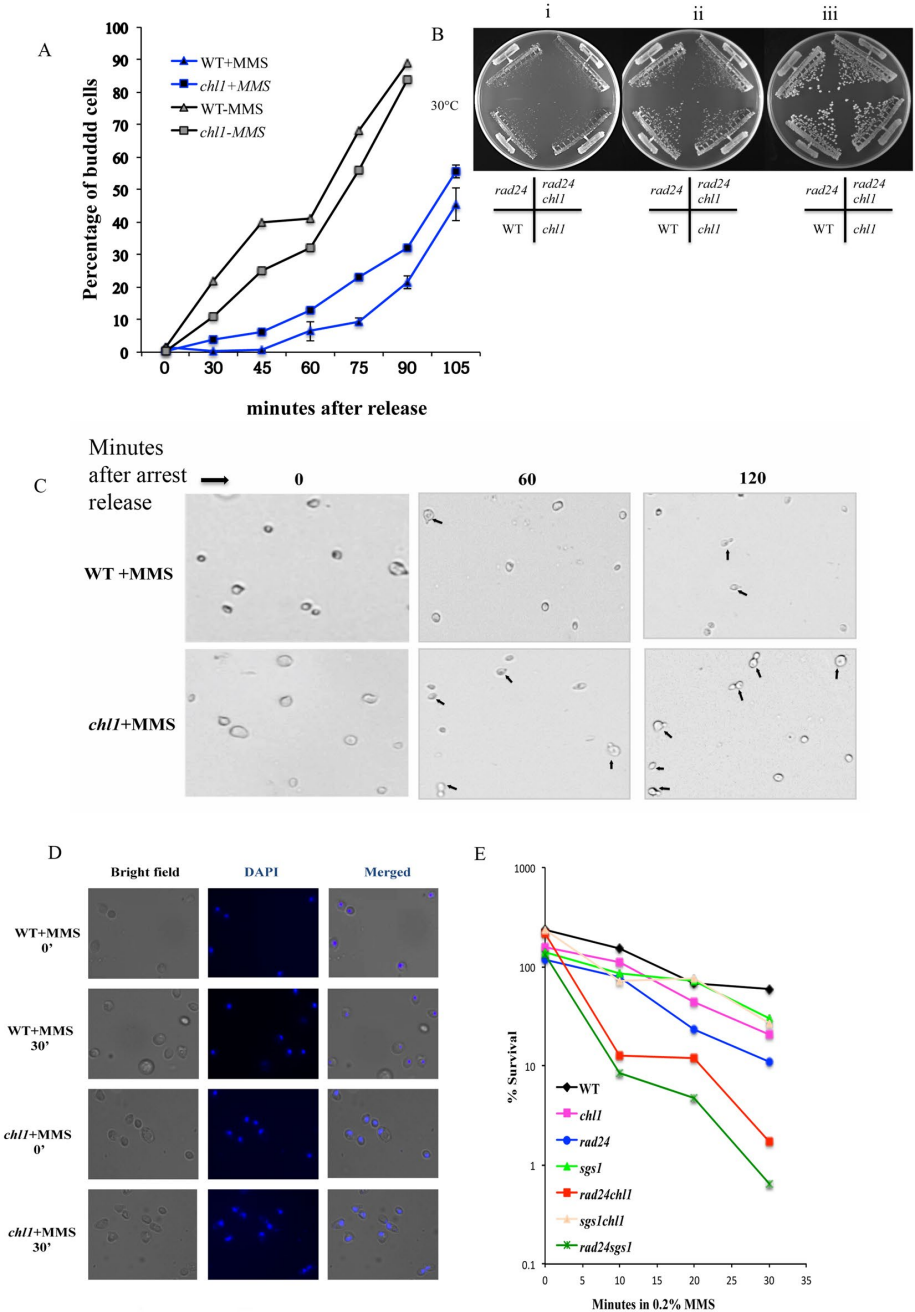
Chl1p is required for G1/S after DNA damage by MMS.** A** *G1-phase bud emergence kinetics of mutant and wild-type cells after MMS treatment.* Wild-type (699) and mutant cell 699Dchl1 (*chl1*) were grown to exponential phase (~ 0.2 OD_610nm_) and arrested with 5 μg/ml *α*-factor for 90 min (G1 arrest) as described in materials and methods. After 80 min of *α*-factor treatment at 30 °C, each culture was divided into two. To one half 0.2% MMS was added and the other was maintained as a control. Cells were kept shaking for a further 10 min. After treatment, MMS was inactivated by the addition of one volume of 10% sodium thiosulfate solution, cells were spun down and the pellet was washed quickly with YEPD medium at RT. The cells were released in a fresh YEPD medium at 30 °C and aliquots were removed at regular times for scoring the percentage of budded cells. The graph represents the percentage of bud emergence in WT and *chl1* cells at different time intervals after release from G1 arrest and 0.2% MMS treatment simultaneously. The black filled symbols are given for cells treated with MMS, the grey filled symbols indicates the absence of MMS. Data shown are averages of values obtained from three independent experiments and the deviations from the mean are shown as error bars. **B** *Growth of WT and mutant cells on YEPD plates.* Wild-type (699) and mutant cell 699Dchl1 (*chl1*), SL3 (*rad24*) and SL3Dchl1 (*rad24chl1*) were streaked for single colony on YEPD plates and incubated at 30 °C for (i) 30 h, (ii) 34 h and (iii) 60 h respectively. We could observe an initial growth difference between the WT and the mutant, *chl1* that goes of after 60 h of incubation **C** *Budding of mutant and wild-type cells after MMS treatment.* The bright fields of WT and *chl1* from (A) at 40X resolution shows the budded cells in wild-type (699) and *chl1* (699DChl1) mutant cultures after 1 and 2 h of release from MMS treatment. The budded cells are indicated with arrows. **D*** Chl1 cells have fragmented DNA at G1 phase when treated with MMS.* 699 (wild-type) and 699Dchl1 (*chl1*) cells were arrested at G1 by treating the log phase cells with alpha-factor for 90 min. To these G1 blocked cells, 0.2% MMS was added to create a substantial damage. Cells were collected at different time points of MMS exposure for DAPI staining. 0’ was collected just after adding 0.2% MMS to the cells with alpha-factor (G1-blocked) followed by 10’, 20’ and 30’ of exposure to 0.2% MMS in presence of alpha-factor (G1-blocked damaged cells). Representative fields of DAPI staining of cells treated for 0’ and 30’ with 0.2%MMS is given for WT and *chl1* mutant cells. The corresponding bright field and merged images are also given along with the DAPI field. **E** *chl1 cells are sensitive towards killing by genotoxic agent in G1/S-phase.* 699 (wild-type), 699Dchl1 (*chl1*), SL3 (*rad24*), SL3Dchl1 (*rad24chl1*), 699Δsgs1 (*sgs1*), 699Δsgs1Dchl1 (*sgs1chl1*) and SL21 (*sgs1rad24*) cells were arrested by alpha-factor in G1. To these G1 blocked cells, 0.2% MMS was added to create a substantial damage at G1. Cells were collected at different time points of MMS exposure for viability assay. 0’ was collected just after adding 0.2% MMS to the cells with alpha-factor (G1-blocked) followed by 10’, 20’ and 30’ of exposure to 0.2% MMS in presence of alpha-factor (G1-blocked damaged cells). Aliquots removed for cell viabilities at the indicated time points were washed off of both MMS and alpha-factor, resuspended in water, counted and plated after dilution on YEPD plates. The plates were incubated at 30 °C for 2–3 days and the viable colonies were counted

Exponentially growing mutant and wild-type cells were arrested in G1 by alpha-factor for 90 min, treated with 0.2% MMS at the last 10 min of arrest and washed free of cell cycle block. MMS was quenched by 10% v/v sodium thiosulphate and released in a fresh medium. Thereafter, at different time intervals, bud emergence was scored as a measure for functional G1/S arrest. The experiment is performed in triplicate with the same time points and nearly 150 cells were counted every time confirming the consistency of the faster bud emergence. The budding kinetics of *chl1* cells is significantly faster than the wild type cells leading us to conclude that Chl1 mutant cells were deficient in G1/S arrest when their DNA was damaged with MMS, (Fig. 1A). There was no significant difference between the WT and *chl1* cells in the kinetics of bud emergence in absence of any MMS treatment (Fig. 1A). Though the budding is slow in the initial time points for *chl1*, it catches up with WT in later time points, which is the normal behaviour of *chl1* cells as shown in Fig. 1B. Budding cells are more in *chl1* mutant cells compared to wild-type cells after 1 and 2 h of MMS treatment as shown by randomly taken representative fields (Fig. 1C). Thus, Chl1p is required for G1/S arrest in response to DNA damage at the G1 phase. The fast movement of chl1 mutants through G1 phase indicates that the cells are spending less time for repair and may have compromised arrest at G1 due to a defective checkpoint. Faster bud emergence due to absence of a halt for repair will lead to increase fragmented DNA. 4',6-Diamidino-2-Phenylindole(DAPI) staining confirms the absence of integrity in the DNA of *chl1* cells when exposed to MMS at G1 block (Fig. 1D). To confirm the defect in G1/S arrest and justifying the progression in cell cycle of the mutant cells with more damage as a result of compromised repair, we performed the sensitivity analysis of *chl1* cells towards the genotoxic agents. Mutant and wild-type cells were arrested in G1 using α-factor for 90 min and then treated with 0.2% MMS. Aliquots of cells exposed to 0.2% MMS in presence of alpha-factor block were taken at various time intervals. Cells were counted and plated on Yeast Extract Peptone Dextrose (YEPD) plates to determine viability. Figure 1E shows nearly 75% loss in the viability of *chl1* cells after 30 min of 0.2% MMS treatment at G1/S. The loss in cell viability of *chl1* compared to wild-type cells in the presence of 0.2% MMS confirmed the accumulation of more damage due to compromised repair and checkpoint molecules.

### Chl1p is not required at G2/M for MMS-induced DNA damage repair

**Fig. 2:**
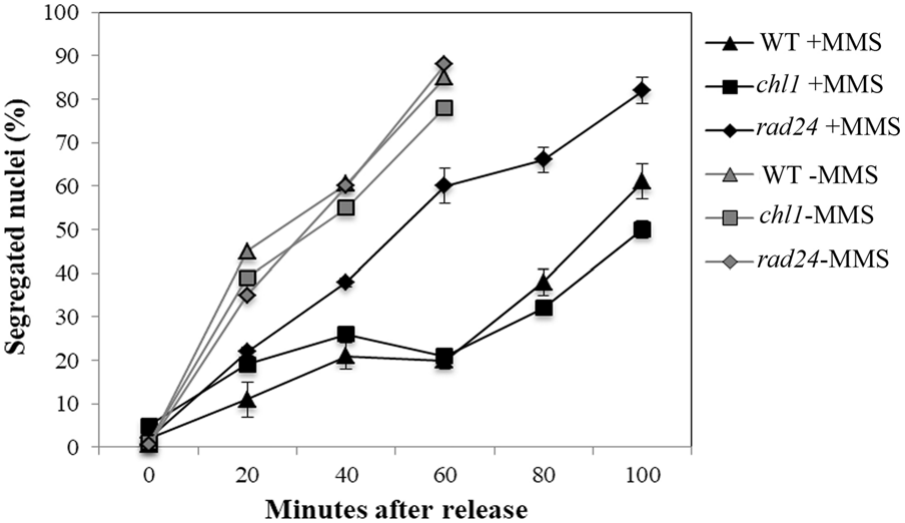
Chl1p is not required for G2/M arrest after DNA damage by MMS. Wild-type (699) and mutant cells 699Dchl1 (*chl1*) and SL3 (*rad24*) were grown to exponential phase and arrested with 15 μg/ml nocodazole for 3 h at 30 °C for G2/M arrest. The arrested cells were treated with 0.15% MMS during last half an hour of nocodazole arrest and kept shaking. After treatment, MMS was inactivated by the addition of one volume of 10% sodium thiosulfate solution. Cells were washed with YEPD medium and released in fresh medium at 30 °C and aliquots were removed at regular intervals and stained with DAPI to score for the percentage of cells with divided nuclei. Data shown are average of values obtained from three independent experiments and error bars are standard deviations from the mean value. The filled symbols are given for cells treated with MMS, the empty symbols indicate the absence of MMS. Error bars are not shown for data points pertaining to minus MMS experiments to avoid cluttering

In presence of DNA damage caused by MMS, G2/M-arrested wild-type cells delay nuclear division [18, 19]. To determine if Chl1p is required in this delay, mutant and wild-type cells were arrested at G2/M by nocodazole, treated with MMS, washed free of cell cycle block including MMS and released into fresh medium. The percentage of cells, which had divided their nuclei, was scored at different time intervals to measure G2/M arrest. Figure 2 shows that *chl1* mutant cells were proficient for G2/M arrest as they delayed nuclear division when their DNA was damaged with MMS. Also, the control cells did not show any significant differences in the timings of nuclear division. Therefore, Chl1p is not required at the G2/M transition for MMS-induced DNA damage repair.

### Chl1p plays a role in regulating the checkpoints at G1/S phase of the cell cycle

The observation that the *chl1* null mutations arrested at G1/S shows sensitivity to genotoxic agents like MMS shows its link with the surveillance mechanism on the genetic stability of the cells. The faster movement of the cells towards bud formation in presence of damage can be an effect of perturbed checkpoint function. As the preliminary observations give a clue of compromised checkpoint function in *chl1* mutant, we decided to confirm this by more direct experiments, as described below.

**Fig. 3:**
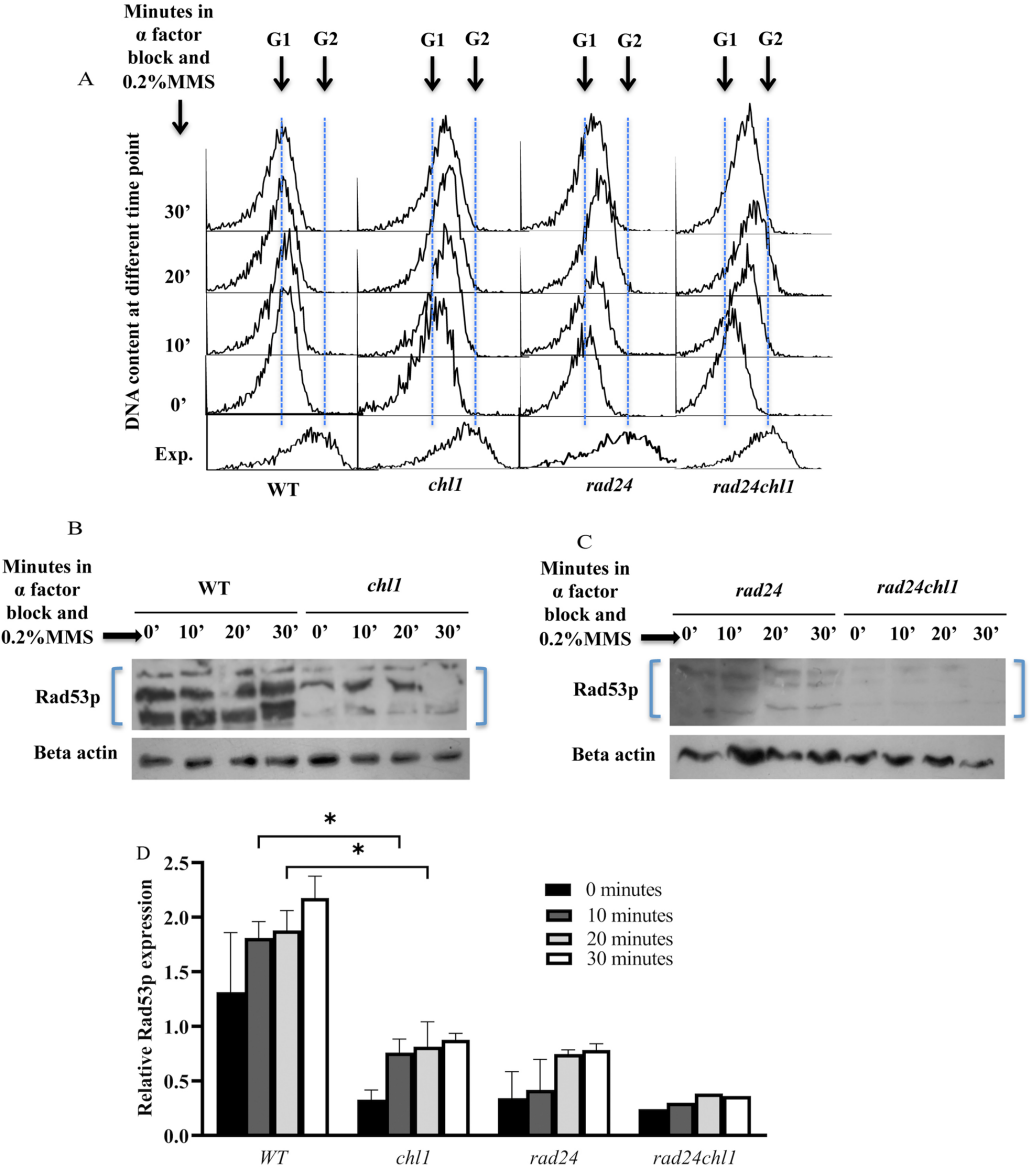
Chl1p plays a role in regulating the checkpoints at G1/S phase of the cell cycle. **A** *G1/S-phase progression of mutant and wild-type cells in the presence of MMS.* Wild-type (699) and mutant cell 699Dchl1 (*chl1*), SL3 (*rad24*) and SL3Dchl1 (*rad24chl1*) were all synchronized with alpha-factor at 30 °C and 0.2% MMS was added in presence of the G1 block. All the cultures were kept shaking at 30 °C. Aliquots were removed at various times for FACS analysis. The histogram plot at each time point are overlayed in the figure by using overlay software to understand the progression of the cells through cell cycle. The exponential cells were collected just before the addition of alpha-factor to the growing cells of 0.2 OD_610nm_. Arrows indicates G1 and G2 DNA contents. **B** *chl1 cells are compromised in Rad53p phosphorylation in response to MMS treatment in G1/S-phase.* Wild type, *CHL1* (699) and 699Dchl1 (*chl1*) cells were arrested in G1 phase and exposed to 0.2% MMS at 30 °C. Rad53p phosphorylation was detected by western blot analysis of proteins extracted from aliquots of cells removed at indicated times, using antibodies directed against the Rad53 protein. **C** *rad24chl1 cells are more compromised in Rad53p phosphorylation compared to chl1 cells in response to MMS treatment in G1/S-phase.* SL3 (*rad24*) and SL3Dchl1 (*rad24chl1*) cells were arrested in G1 phase and exposed to 0.2% MMS at 30 °C along with the cells of **B**. Rad53p phosphorylation was detected by western blot analysis of proteins extracted from aliquots of cells removed at indicated times, using antibodies directed against the Rad53 protein. **D** *Quantification of Rad53p expression in chl1 cells along with the double mutant rad24chl1 cells.* The intensity of the phosphorylated bands of Rad53p in WT (*CHL1*), 699Dchl1 (*chl1*), SL3 (*rad24*) and SL3Dchl1 (*rad24chl1*) cells in western blots was quantified using Image J software. The values of the Rad53p phosphorylated band intensities taken together were normalized with corresponding intensities of beta-actin to normalize the protein loading at different time points. The 0 min is just after adding 0.2% MMS followed by 10, 20 and 30 min exposure to 0.2% MMS. The graph shows the average of data obtained from 3 repeated experiments

The checkpoint kinase proteins inhibit the cell cycle progression in presence of damage, allowing time for DNA repair to take place [16, 39]. However, when DNA is damaged in G1/S or S-phase checkpoint mutants such as *mec1, rad9, rad17, rad24* and *rad53*, S-phase appears to progress faster because of inappropriate initiation of the origins, causing additional DNA synthesis, which can be detected by flow cytometry [16, 39]. To test whether Chl1p affects the G1/S phase checkpoint function, the progression of cell cycle at G1/S was observed by monitoring the DNA content through flow-cytometry in 0.2%MMS treated G1 synchronized cells. Once the cells reaches G1 upon alpha-factor treatment for 90 min, the cells were exposed to 0.2%MMS without releasing from alpha-factor and the progression of DNA synthesis from G1 to S was monitored by flow cytometry. The *chl1* cells came out from G1 arrest by 10 min of treatment with MMS in presence of alpha-factor whereas in case of wild type the entry in *S*-phase from G1 was not observed (Fig. 3A). Since the G1 to S-phase progression in *chl1* was faster compared to wild-type cells in the presence of high MMS damage, it suggests that the DNA damage checkpoint pathway is perturbed in these cells. We also observed the faster movement of DNA from G1 to S phase in the known DNA damage checkpoint mutant *rad24.* Interestingly the double mutant *rad24chl1* moved fastest confirming the synergistic role of both Chl1p and Rad24p as checkpoints in presence of damage suggesting that they may follow two parallel pathways (Fig. 3A). The checkpoint mutant *sgs1* were also included along with *rad24* in the cell cycle progression studies as they have roles in replication checkpoint, like *rad24* in damage checkpoint pathways [40, 41]. In case of replication checkpoint, *sgs1* cells showed progression like WT and Sgs1Chl1 mutant was not significantly different from *sgs1* (Additional file 1: Fig. S1). The slow progression of *sgs1* like WT is because of the absence of any role of Sgs1p at G1 and also the presence of a functional repair mechanism. Sgs1 mutants halt for repair like WT at G1 in presence of damage. But in Chl1 mutants, due to defect in repair and checkpoint, it progresses faster in cell cycle in presence of damage. In case of *sgs1chl1* and *sgs1rad24* the progression is similar to chl1 mutants and Rad24 mutants respectively and Sgs1 mutation plays no additive role in them.

To confirm the effect of Chl1p on checkpoints at G1/S phase, Rad53p activation was compared between wild type and *chl1* mutant cells by directly assaying for its phosphorylation in MMS-treated G1 arrested cells. Cells were synchronized with alpha-factor and treated with 0.2%MMS once all the cells reached the G1 phase. Aliquots were withdrawn at indicated times. Figure 3B and D shows that *chl1* cells had compromised Rad53p phosphorylation and which is significantly low by 10’ of 0.2% MMS exposure compared to the wild type at G1/S-phase. Thus, this confirms that Chl1p is required to activate the DNA damage checkpoint pathway when cells are treated with MMS in G1/S-phase.

To further confirm that Chl1p acts in parallel to the damage checkpoint pathway, we monitored Rad53p phosphorylation both in WT, single checkpoint mutants and checkpoint mutants along with *chl1* at G1/S phase in presence of 0.2% MMS. The checkpoint mutant *rad24* was included in the Rad53p phosphorylation studies as it has a role in the damage checkpoint pathway [40, 41]. *rad24* cells, as expected, showed lower levels of Rad53p phosphorylation (Fig. 3C, D). Interestingly Rad24Chl1 mutant was even more compromised in phosphorylating Rad53p than *rad24* and *chl1* alone (Fig. 3C, D). We thus observed that the *chl1* cells started coming out from G1 arrest faster like the *rad24* checkpoint mutant cells in presence of 0.2% MMS treatment to G1 arrested cells in just 10 min. We also observed a compromised checkpoint activity of Rad53p in absence of Chl1p. The double mutant *rad24chl1* was even faster in coming out from arrest with a broader peak and had further reduced Rad53p activity. So in this section, we confirmed the role of Chl1p, in addition to Rad24p, in regulating the checkpoint pathway through Rad53p activation in G1/S.

### Chl1p acts independently of the DNA damage checkpoint pathway

**Fig. 4:**
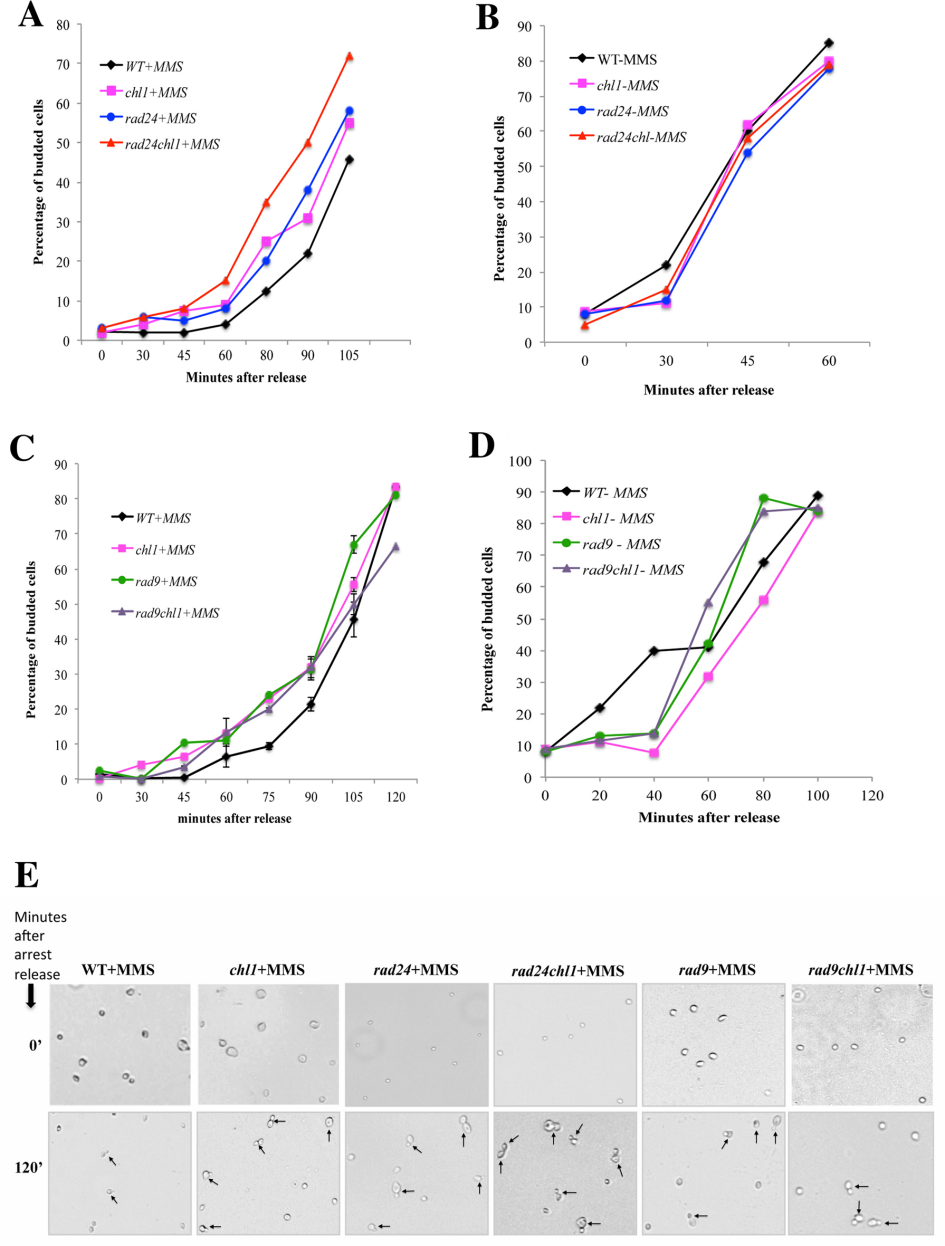
Chl1p acts independently of the DNA damage checkpoint pathway.** A** *G1-phase bud emergence kinetics of Chl1 mutant cells are additive to rad24 after MMS treatment.* Wild-type (699) and mutant cells 699Dchl1 (*chl1*), SL3 (*rad24*), SL3Dchl1 (*rad24chl1*) were grown to exponential phase and arrested with 5 μg/ml *α*-factor for 90 min (G1 arrest) as described in materials and methods. After 80 min of *α*-factor treatment at 30 °C, 0.2% MMS was added. Cells were kept shaking for a further 10 min. After treatment, MMS was inactivated by the addition of one volume of 10% sodium thiosulfate solution, cells were spun down and the pellet was washed quickly with YEPD medium at RT. The cells were released in a fresh YEPD medium at 30 °C and aliquots were removed at regular times for scoring the percentage of budded cells. The graph represents the percentage of bud emergence in WT, *chl1, rad24 and rad24chl1* cells at different time intervals after release from G1 arrest and 0.2% MMS treatment simultaneously. Data shown are the average of values obtained from three independent experiments. **B** *G1-phase bud emergence kinetics of cells in absence of MMS treatment.* Wild-type (699) and mutant cells 699Dchl1 (*chl1*), SL3 (*rad24*), SL3Dchl1 (*rad24chl1*) were simultaneously grown with the **A** cells to exponential phase and arrested with 5 μg/ml *α*-factor for 90 min (G1 arrest). The cells were released in a fresh YEPD medium without any MMS treatment at 30 °C and aliquots were removed at regular times for scoring the percentage of budded cells. **C** *G1-phase bud emergence kinetics of Chl1 mutant cells is in line with rad9 after MMS treatment.* Wild-type (699) and mutant cells 699Dchl1 (*chl1*), SL19 (*rad9*), SL19Dchl1 (*rad9chl1*) were grown to exponential phase and follow through same experimental procedures as done in **A**. The graph represents the percentage of bud emergence in WT, *chl1, rad9 and rad9chl1* cells at different time intervals after release from G1 arrest and 0.2% MMS treatment simultaneously. Data shown are average of values obtained from three independent experiments. **D** *G1-phase bud emergence kinetics of cells in absence of MMS treatment.* Wild-type (699) and mutant cells 699Dchl1 (*chl1*), SL19 (*rad9*), SL19Dchl1 (*rad9chl1*) were simultaneously grown with the **C** cells to exponential phase and arrested with 5 μg/ml *α*-factor for 90 min (G1 arrest). The cells were released in a fresh YEPD medium without any MMS treatment at 30 °C and aliquots were removed at regular times for scoring the percentage of budded cells. **E** *Additive and synergistic budding of mutant and wild-type cells after MMS treatment.* The bright fields of WT and mutant cells from (**A**, **B**) at 40X resolution shows the budded cells in wild-type (699) and the mutant cells 699Dchl1 (*chl1*), SL3 (*rad24*), SL3Dchl1 (*rad24chl1*), SL19 (*rad9*), SL19Dchl1 (*rad9 chl1*) mutant cultures after 2 h release from MMS treatment. The budded cells are indicated with arrows

The sensitivity of *chl1* cells and damage of DNA as shown by DAPI towards xenotoxic agents, faster movement through the cell cycle in presence of damage at G1/S and compromised Rad53 activity proves the perturbed checkpoint functioning at G1 in *chl1* mutant cells. Further cell cycle progression studies with damage checkpoints and replication checkpoints confirm it to be additive to damage checkpoints rather than replication checkpoints. To confirm the pathway analysis of Chl1p’s checkpoint activity on the budding kinetics we performed the following experiments. The intra-S-phase checkpoint proteins Sgs1 and Rad24 act in parallel in the DNA replication and damage checkpoint pathways, respectively to maintain the genomic integrity. They maintain cell viability and activate Rad53p in the presence of damage through genotoxic agents [19, 42]. In the viability studies, the single mutants *sgs1* and *rad24* were included along with *chl1*. The double mutants *rad24chl1* and *sgs1chl1* were also included to determine if *chl1* showed any synergistic loss in viability with either of these two mutations at G1/S in 0.2% MMS. The results (Fig. 1E) show that there is a synergistic drop in cell viability in *rad24chl1* double mutants but not in *sgs1chl1*. The *rad24sgs1* double mutant exhibited an expected fall in cell viability. This shows that Chl1 acts in addition to the Rad24 pathway. To further confirm the pathway of Chl1p for G1- arrest we performed the bud emergence experiments with mutant genes, which regulates the effect of genetic insults on cell cycle kinetics, like *rad9*, *rad24* and the corresponding double mutants at G1. Rad9 and Rad24 epistasis group are required for efficient cell-cycle arrest after DNA damage in G1/S [12, 13] and *G2*/*M* [19, 43]. To determine if Chl1p is in Rad9p or Rad24p pathway at this phase of the cell cycle, experiments were carried out to monitor the kinetics of bud emergence. WT, *chl1*, *rad9*, *rad24*, *rad24chl1* and *rad9chl1* cells were arrested in G1 by alpha-factor, treated with 0.2% MMS, washed free of cell cycle block and MMS, and released into fresh medium to score for bud emergence. Figure 4A shows that the double mutant *rad24chl1* emerged from the arrest faster than either of the single mutants *chl1* and *rad24* and the effect appeared to be additive with *chl1* mutation. This confirms that Chl1p acts independently of Rad24p to arrest damaged cells at G1/S phase. In absence of MMS we found no significant difference in the budding kinetics after release from G1 block between *chl1*, *rad24* and *rad24chl1* cells compared to WT (Fig. 4B). On contrary to *rad24chl1* budding kinetics, Fig. 4C shows that the double mutant *rad9chl1* doesn’t emerge from the arrest any faster than either of the single mutants, *rad9* and *chl1*. Thus, Chl1p acts through the Rad9 pathway. The bud emergence of the same strains also shows no significant difference compared to WT in absence of any DNA insult (Fig. 4D). Representative fields of budding cells of the single mutants *rad24*, *rad9* and the double mutants *rad24chl1*, *rad9chl1* also proves that *chl1* mutant cells have more buds compared to wild-type cells after 2 h of MMS treatment and the number of buds in case of *rad24chl1* is significantly more compared to *rad24* and *chl1* alone (Fig. 4E).

### Chl1p plays a dual role in the mode of arrest upon DNA damage in G1/S phase of the cell cycle

The pathway analysis (shown in Fig. 4), the sensitivity studies towards genotoxic agents (as shown in Fig. 1) and compromised Rad53p activity (Fig. 3) of chl1 mutants suggests that Chl1p has a role in regulating checkpoints and acts in a synergistic way to the DNA damage checkpoint pathway. But, faster kinetics of bud emergence compared to the wild-type can also suggest that Chl1p could be involved in damage repair, and in absence of it the cells escape the time to repair the damage and hence moves faster towards budding.

**Fig. 5:**
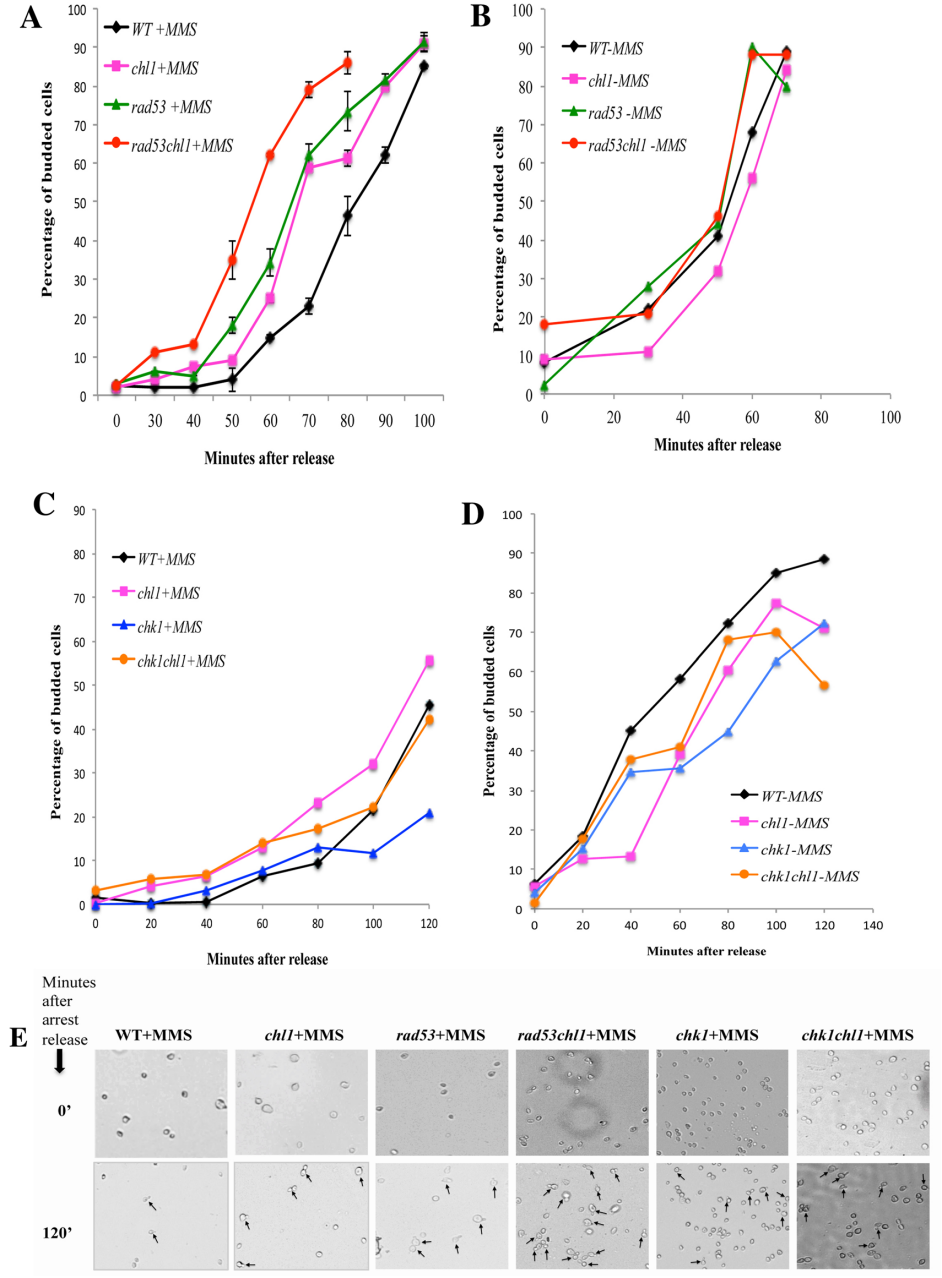
Chl1p plays role in dual mode of arrest upon DNA damage in the G1/S phase of the cell cycle.** A** *Chl1p acts independently of Rad53p at G1/S after DNA damage.* Wild-type (699) and mutant cells 699Dchl1 (*chl1*), SL7 (*rad53*) and SL7∆chl1 (*rad53chl1*) were grown to exponential phase and follow through same experimental procedures as done in 4A. The graph represents the percentage of bud emergence in WT, *chl1, rad53 and rad53chl1* cells at different time intervals after release from G1 arrest and 0.2% MMS treatment simultaneously. Data shown are averages of values obtained from three independent experiments and the deviations from the mean are shown as error bars. **B** *G1-phase bud emergence kinetics of cells in absence of MMS treatment.* Wild-type (699) and mutant cells 699Dchl1 (*chl1*), SL7 (*rad53*) and SL7∆chl1 (*rad53chl1*) were simultaneously grown with Fig. 5A cells to exponential phase and arrested with 5 μg/ml *α*-factor for 90 min (G1 arrest). The cells were released in a fresh YEPD medium without any MMS treatment at 30 °C and aliquots were removed at regular times for scoring the percentage of budded cells. **C** *Chk1p plays no role at G1/S after MMS treatment.* Wild-type (699) and mutant cells 699Dchl1 (*chl1*), SL26 (*chk1*) and SL27 (*chk1chl1*) were grown to exponential phase and follow through the same experimental procedures as done in 4A. The graph represents the percentage of bud emergence in WT, *chl1, chk1 and chk1chl1* cells at different time intervals after release from G1 arrest and 0.2% MMS treatment simultaneously. The bud emergence kinetics of *chk1* is similar to WT and *chk1chl1* is similar to the bud emergence kinetics of *chl1*. Data shown are average of values obtained from three independent experiments. **D** *G1-phase bud emergence kinetics of cells in absence of MMS treatment.* Wild-type (699) and mutant cells 699Dchl1 (*chl1*), SL26 (*chk1*) and SL27 (*chk1chl1*) were simultaneously grown with the Fig. 5C cells to exponential phase and arrested with 5 μg/ml *α*-factor for 90 min (G1 arrest). The cells were released in a fresh YEPD medium without any MMS treatment at 30 °C and aliquots were removed at regular times for scoring the percentage of budded cells. **E** *Budding of mutant and wild-type cells after MMS treatment.* The bright fields of WT and mutant cells from (**A** and **C**) at 40X resolution show the budded cells in different cultures after 2 h release from MMS treatment. The budded cells are indicated with arrows

Earlier we have shown that in S-phase, Chl1p plays a role in the repair pathway upon DNA damage [7]. As Chl1p acts as a repair protein in S-phase, we wanted to determine if Chl1p has some additional role at G1 phase in addition to regulating Rad53p checkpoint pathway in delaying bud emergence when exposed to damage. To reveal the additional roles of Chl1 we performed the bud emergence experiments with mutant genes *rad53*, *chl1* and the corresponding double mutants. WT, *chl1*, *rad53* and *rad53chl1* cells were arrested in G1 by alpha-factor, treated with 0.2% MMS, washed free of cell cycle block and MMS, and released into fresh medium to score for bud emergence. Figure 5A shows that the single mutants are faster than the WT and the double mutant *rad53chl1* emerges significantly faster from G1 arrest than the single mutants *chl1* and *rad53*. Figure 5B shows no significant difference in the budding kinetics of the same cells in absence of MMS. The randomly captured representative fields of budding cells of *chl1*, *rad53* and *rad53chl1* also confirm the same (Fig. 5C). The faster bud emergence of the single mutants from WT confirms the checkpoint defect in the single mutant. But the even faster movement of the double mutant *rad53chl1* interestingly suggests that Chl1p may be following a parallel pathway for arresting cells at G1 along with Rad53p checkpoint arrest to maintain the genomic integrity on exposure to different types of genomic insults. The increase of fragmented DNA in *chl1* cells (Fig. 1D, Table 1) compared to WT also confirms the role of Chl1p in DNA repair. Literature suggests that the Chk1 checkpoint pathway acts in parallel to the Rad53p checkpoint pathway in presence of damage at G2 and M phases [34, 35]. Also, this DNA checkpoint kinase phosphorylates after MMS treatment in a Rad9-dependent and Rad53-independent manner [36]. As per our bud emergence data we can confirm that Chl1p follows a pathway in addition to Rad53p and Rad24p and goes along with Rad9p. So, Chl1p may regulate both the Rad53p and Chk1p checkpoint pathways at G1. To confirm this hypothesis we checked whether Chk1p has any role at G1 and is it linked with Chl1p. We studied the budding kinetics of single mutants *chl1* and *chk1* along with the double mutant *chl1chk1* (Fig. 5D). Though the bud emergence of *chl1* mutant’s was faster than the WT, there was no additional difference of bud emergence of *chk1chl1* from *chk1* and *chl1*. Also, the budding of *chk1* cells was similar to WT. All these observations prove that Chk1p doesn’t play a role in arresting at *G1* in presence of damage and Chl1p doesn’t act through the Chk1p pathway. The budding kinetics of WT, *chl1*, *chk1* and *chl1chk1* were almost the same in absence of any damage (Fig. 5E). So, in this section we prove that Chl1p regulates two pathways in G1 phase to delay bud emergence in presence of damage, one is through Rad53p by modulating its phosphorylation and the other one is parallel to Rad53p but doesn’t follow the Chk1p checkpoint pathway. So, the other pathway in which Chl1p has some role is the damage repair in G1 (role in repair in S phase is already known).

**Table 1:** The MMS treated cells showing percentage of DNA damage at 0 and 30 min in wild type and mutant strains

Strains	MMS (Minutes’)	Cells with compact DNA (%)	Cells with fragmented DNA (%)
699	0’	95.49	4.505
	30’	88.17	11.83
699chl1	0’	92.86	7.14
	30’	27.39	72.61

## Discussion

The functioning of Rad9p as G1/S checkpoint is dependent on its TUDOR and BRCT domains and is independent of its auto-phosphorylation through CDK [44]. Rad53p activation in G1 and S phase depends on the association of Rad9p with the modified chromatin adjacent to Double Standard Break (DSBs). Rad9p-chromatin association is mediated by the binding of TUDOR domains to histone di-methylated H3 and BRCT domains binding to phosphorylated histone H2A [37]. If the interaction is broken the activation of phosphorylated Rad53 is compromised in presence of a genotoxic agent like MMS and Hydroxyurea (HU). The *RAD9* BRCT mutant fails to perform the G1 checkpoint delay post DNA insult but it was proficient in checkpoint response upon DNA damage in nocodazole treated cells. So, the recruitment and retention of Rad9p at the damage sites through the BRCT domain play a vital role in the G1/S arrest. The interactor proteins of Rad9p at the BRCT domain are also instrumental in maintaining the arrest for proper repair of the damage. The human homolog of Chl1p is BACH1 and that for Rad9p is BRCA1. In mammalian system, at *G1*-phase, BACH1 is phosphorylated leading to the interaction with BRCA complex through BRCT domain, with low Adenosine Triphosphatase (ATPase) /helicase activity. As a result, the movement of the replication complex slows down enhancing the proof reading activity of the polymerase. Adversely, during the slow down of the fork, the nascent leading and lagging strands tend to anneal to each other due to fork regression or reversal to form secondary structures [34]. The complex of BACH1/BRCA along with the combination of BLM1, a helicase with opposite polarity, resolves these difficult structural motifs encountered by the replication forks during DNA replication [45]. Once the proofreading and resolving activity of the secondary structures are over, the de-phosphorylation of BACH1 takes place. On de-phosphorylation, the BACH1/BRCA complex breaks down, leaving behind BACH1 at the fork generating the space for the replication machinery to start replication [45]. Simultaneously dephosphorylated BACH1 regains the helicase activity to unwind the DNA for timely progression through S-phase. So looking at the correlation and domain analogy of BACH1 and BRCA1 in mammalian system it can be concluded that Chl1p binds to Rad9p through the BRCT domain and allows Rad9p to sense the damage because of its repair and helicase activity. So, most probably the retention of Rad9p at the damage site is because of its BRCT interactor Chl1p. The recruited Rad9p activates the checkpoint Rad53p to bring in the cell cycle arrest and Chl1p gets the time to repair the damage.

**Fig. 6:**
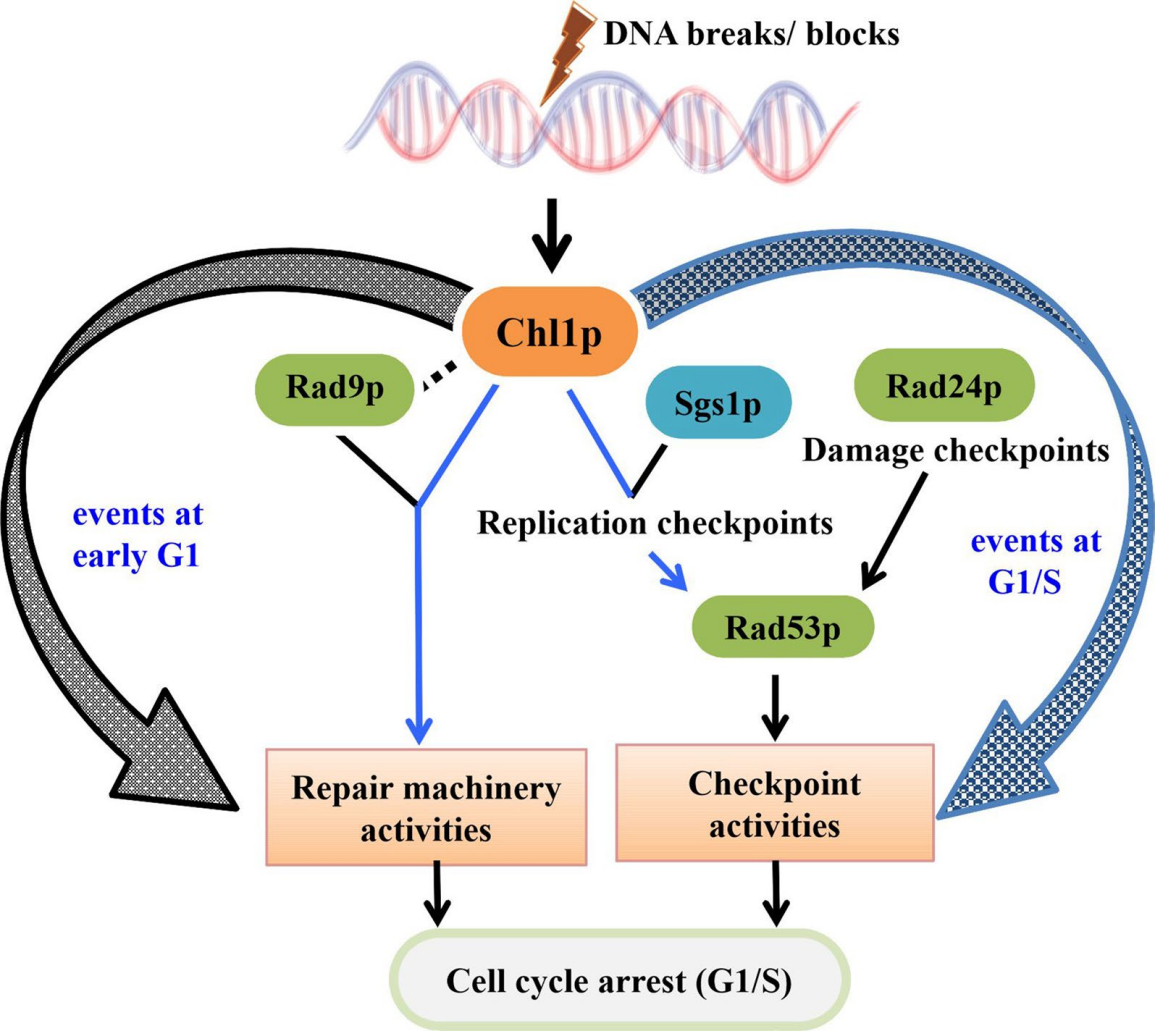
Roles of Chl1p at G1 phase of the cell cycle of budding yeast. The schematic representation depicts the multifunctional role of Chl1p at G1, once the DNA faces any insults with genotoxic agents. The wide curved grey arrow indicates the event regulated by Chl1p at early G1 phase and the blue curved one points towards the function played at G1/S. The black lines and the arrows indicate the known pathways. The blue lines and the arrows are the proposed associations explained in this paper with supporting observations. The black dotted line shows the plausible association between the two proteins, Rad9 and Chl1, which is already present in their human homologs (BRCA1 and BACH1 respectively)

In this paper, we show evidence that, like *rad9*, chl1 mutants also fail to execute the G1 checkpoints and the delay in bud emergence is perturbed in G1-arrested cells when treated with MMS. In the presence of damage, Chl1p executes the G1/S phase arrest. In chl1 mutants, faster kinetics of bud emergence compared to the wild-type, additionally, faster budding of *rad53chl1* cells compared to *chl1* and *rad53* suggests that Chl1p could be involved in repair, and in absence of it, the cells escape the time to repair the damage and hence moves faster towards budding with more accumulated damage and sensitive towards MMS. Compromised Rad53 activity of *chl1* cells at G1 in presence of MMS damage confirms its other role in regulating checkpoint pathway which also adds up in maintaining the budding kinetics at G1 after DNA damage with 0.2% MMS. It plays the checkpoint role parallel to the damage checkpoint pathway in G1 phase of the cell cycle as the Rad53p phosphorylation of chl1 mutants is even more compromised in absence of *rad24.* The checkpoint role through Rad53p and not through Chk1p, and the repair function in addition to Rad53 phosphorylation of Chl1p regulates the G1 phase arrest when DNA is perturbed. So Chl1p plays a role in regulating checkpoint at G1/S phase, which leads to Rad53p activation through Rad9p and prevents bulk DNA synthesis. It regulates the repair function in addition, which is independent of Rad53p and in synchrony with Rad9p to regulate the budding kinetics following insult to the genetic material. So, in a nutshell Chl1p plays multiple roles throughout the G1 phase of the cell cycle as presented in the schematic representation of Chl1p involving pathways at G1 (Fig. 6). G2/M phase arrest is executed by the auto-phosphorylation of Rad9p and is independent of the BRCT domain [46]. Establishment of sister chromatid cohesion occurs for the repair of double strand breaks at G2/M [47, 48]. Since Chl1p is required for the establishment of sister chromatid cohesion [1], resistance of *chl1* mutant towards faster budding kinetics and killing by MMS treatment at G2/M suggests that the repair of this damage is not critically dependent on the cohesion function of Chl1p.

## Conclusion

In summary, this paper brings to light additional role of cell cycle regulation by Chl1p in budding yeast. In presence of Chl1p, the repair and checkpoint functions are proficient in cells with double strand breaks, and so able to perform the G1/S delay in bud emergence. Chl1p leads to Rad53 activation, the major effector checkpoint kinase in presence of damage at *1*. The Rad53p checkpoint activation by Chl1p at G1/S is independent of the Rad24p mediated damage checkpoint pathway. We also show that the role of Chl1p for bud emergence in G1 phase is in line with Rad9p and independent of Rad24p/ Rad53p. Sgs1p and Chk1p seem to play no role in G1 and the function of Chl1p doesn’t associate with them. The, double mutant *rad9ch1* and *chk1chl1* shows similar bud emergence as the single mutants *chl1*, *rad9* and *chk1* whereas the double mutant *rad24chl1* and *rad53chl1* shows faster bud emergence than the single mutants. This budding kinetics explains an additional role of Chl1p independent of Rad53p checkpoint activation. This paper supports a model in which Chl1p plays a critical role in regulating the G1/S transition along with Rad9p when cells are compromised with DNA damaging agents. Consistent with our data and the supporting experimental findings from other groups, we predict that the helicase Chl1p plays a role in modulating the chromatin structure of the damaged DNA, aids Rad9p BRCT domain to access phosphorylated H2A S129 residue at the double strand break region followed by engagement of repair machinery. The repair process is further supported by the checkpoint activation through Chl1p. The checkpoint property further activates downstream regulators and key checkpoint proteins and keeps the cells arrested at early G1 as well as G1/S transition to provide some time for proper repair of the perturbed DNA at DSBs or blocks.

As the mammalian homologs of Rad9p (BRCA1) and Chl1p (BACH1) interacts at the BRCT domain [10], helicase Chl1p is suspected to be the Rad9p interactor and presumed to play the role of repair and remodeling of the damaged DNA along with Rad9p at the damaged sites. The findings of this paper gives a clue that the association of Rad9p to the modified chromatin at the DSB’s helps to bring Chl1p repair protein through interaction with BRCT domain and repair damage by delaying G1 to S transition. During damage, the interaction between BRCT domain of Rad9p and phospho-H2A brings in the repair protein Chl1p helicase to the proximity of the damaged sites. As Chl1p also acts as a chromatin-remodeling factor [6], this in turn helps to remodel the chromatin bound Rad9p and initiate repair activity by arresting the cells at G1. The G1/S phase arrest is further supported by its Rad53p dependent checkpoint activity.

## Materials and methods

### Media and chemicals

All media, chemicals and enzymes have been described before [7, 12, 49]. DAPI, alpha-factor and goat anti-rat AP-conjugated antibody were from Sigma. Goat anti-mouse TRITC-conjugated antibody and NBT/BCIP was from Bangalore Genei Pvt. Ltd. Rad53 goat polyclonal antibody, raised against a carboxy terminus peptide of yeast Rad53p was from Abcam, and secondary HRP-conjugated anti-mouse antibody was from CST, USA. MMS was from Sigma.

### Construction of single and double mutant strains

Gene disruptions and deletions of Chl1 are described in [50]. Construction of double mutants and PCR based deletion of *CHL1* and *BAR1* were carried out as described in [7, 51]. 699 and all the strains listed in Table 2 are in W303 background while the parent strains of the remaining were from G. Fink.

**Table 2:** lists the strains used for this study

Strain	Genotype	Reference
699	*MAT***a***ade2-1 trp1-1 leu2-3, 112 his 3–11, 15 ura3 can1-100*	[7]
699Dchl1	*MAT***a***ade2-1 trp1-1 leu2-3, 112 his 3–11, 15 ura3 can1-100 chl1::HIS3*	[7]
US456	*MAT***α***leu2 his3 trp1 ade2 rad24::URA3*	Uttam Surana
SL1	*MAT***α***leu2 his3 trp1 ade2 rad24::URA3 chl1::HIS3*	This study, by crossing US456 with 699Dchl1
SL3	*MAT***a***leu2 his3 trp1 ade2 rad24::URA3*	By crossing US456 with 699Dchl1
SL4	*MAT***a***leu2 his3 trp1 ade2 rad24::URA3* *chl1::HIS3*	By crossing US456 with 699Dchl1
US355	*MAT***α***cdc13 rad9 leu2 ura3*	Uttam Surana
SL9	*MAT***a***leu2 his3 trp1 ade2 ura3 rad9*	By crossing US355 with 699
SL9DChl1	*MAT***a***leu2 his3 trp1 ade2 ura3 rad9 chl1::HIS3*	This study, by disrupting *CHL1* in SL9
US354	*MAT***α***leu2 his3 trp1 ade2 ura3 rad53-21*	[7]
SL7	*MAT***a***leu2 his3 trp1 ade2 ura3 rad53-21*	[7]
SL7Dchl1	*MAT***a***leu2 his3 trp1 ade2 ura3 rad53-21 chl1D::TRP1*	This study, by deleting *CHL1* in SL7
699Δsgs1	*MAT***a***ade2-1 trp1-1 leu2-3, 112 his 3-11,15 ura3 can1-100 sgs1Δ::LEU2*	This study, by deleting *SGS1* in 699
699Δsgs1 Dchl1	*MAT***a***ade2-1 trp1-1 leu2-3, 112 his 311,15 ura3 can1-100 sgs1Δ::LEU2 chl1::HIS3*	This study, by disrupting *CHL1* in 699Δsgs1
SL21	*MAT***a***ade2-1 trp1-1 his 3-11,15 ura3 can1-100 sgs1Δ::LEU2 rad24::URA3*	This study, by crossing SL1with 699Δsgs1
SL 26	*MAT***a***ade2-1 trp1-1 leu2-3, 112 his 3-11, 15 ura3 can1-100 Chk1::LEU2*	This study, by deleting *CHK1* in 699
SL27	*MAT***a***ade2-1 trp1-1 leu2-3, 112 his 311, 15 ura3 can1-100 chl1::HIS3 Chk1::LEU2*	This study, by deleting *CHK1* in 699Dchl1

699 and all the strains listed are in W303 background

### Cell synchronization, bud emergence and nuclear segregation

Cells were synchronized in G1 using alpha-factor as described in [52]. Briefly, log phase cells were arrested with 0.025 μg/ml α-factor for 90 min and treated with 0.2% MMS in the last 10 min of arrest at 30 °C. MMS was quenched by 10% v/v sodium thiosulphate. Cells were washed free of cell cycle block (α-factor) and released into fresh medium. Thereafter, at different time intervals bud emergence post DNA damage was scored as a measure of G1/S arrest [53].

For G2/M arrest exponentially growing cells were treated with 15 μg/ml nocodazole for 3 h at 30 °C. The arrested cells were treated with 0.15% MMS during last half-hour of nocodazole arrest. After treatment, MMS was quenched with 10% sodium thiosulfate (v/v) and released from block. Nuclear stain was done with DAPI [54]. Around 150–200 cells were counted for nuclear morphologies, using a fluorescence microscope (Leica fitted with DC 300F camera).

### Flow cytometry

The phases of the cell cycle were determined by flow cytometry according to the protocol described in 12. Briefly, exponentially growing 1–2 X 10^7^ cells were arrested at G1 using alpha-factor. To the arrested cells 0.2% MMS was added. Cells were collected at different time intervals in chilled 70% ethanol to do the cell cycle analysis. The cells fixed from each time point including the exponentials were spun down and fixed overnight in 70% ethanol at 4˚C. Cells were washed and suspended in Tris–EDTA (pH 7.5) buffer for RNaseA treatment at 37 °C for 4 h. Propidium Iodide (50 μg/ml) staining was done overnight at 4 °C. Flow cytometry was done in FACS caliber (Becton Dickinson) with the sonicated samples (10 amps for 15 s).

### Protein extractions and western blot analysis

For western blot analysis, protein extracts were prepared according to [7, 10] from cells synchronized at G1 and treated with 0.2% MMS. Proteins were separated on 8% SDS–PAGE containing an acrylamide to bis-acrylamide ratio of 80:1 and transferred to poly-vinylidene difluoride (PVDF) membrane (Millipore). Rad53 was detected using anti-Rad53 goat polyclonal antibody at 1:1000 dilution in TBS (50 mM Tris buffer pH 7.5, 150 mM NaCl) containing 0.5% BSA for 12–16 h. Secondary alkaline phosphatase-conjugated anti-goat antibody was incubated with the membrane for 2 h at 1:2500 dilution.

## Supplementary Information

Additional file 1: **Fig. S1. **Chl1p follows the replication checkpoint pathway*.*** A ***G1/S-phase progression of single mutant and double mutant cells in the presence of MMS.* Mutant cell 699∆sgs1 (*sgs1*) and the double mutants, SL21 (*rad24sgs1*) and 699∆sgs1Dchl1 (*sgs1chl1*) were arrested at G1 by treating the log phase cells with alpha factor for 90 min at 30 °C. 0.2% MMS was added in presence of the G1 block. All the cultures were kept shaking at 30 °C. Aliquots were removed at various times of MMS exposure for FACS analysis. 0’ was collected just after adding 0.2% MMS to the cells with alpha factor (G1-blocked) followed by 10’, 20’ and 30’ of exposure to 0.2% MMS in presence of alpha factor (G1-blocked damaged cells). The histogram plots at each time point are overlayed in the figure by using overlay software (Guava-Incyte) to understand the progression of the cells through cell cycle. The exponential cells were collected just before addition of alpha factor to the growing cells of 0.2 OD_610nm_. Arrows indicate G1 and G2 DNA contents.

## Data Availability

The datasets used and/or analyzed during the current study are available from the corresponding author on reasonable request.

## Abbreviations

ATP: Adenosine Triphosphate
BACH1: Breast Cancer Associated C-Terminal Helicase 1
BRCT: BRCA1 C Terminus
CDK: Cyclin Dependent Kinase
DAPI: 4',6-Diamidino-2-Phenylindole
DDC1: DNA Damage Checkpoint Protein 1
DSBs: Double Standard Break
HU: Hydroxy Urea
Mec: Mitosis Entry Checkpoint
MMS: Methyl Methane Sulfonate
rDNA: Recombinant DNA
RFC: Replication Factor C
PCNA: Proliferating Cell Nuclear Antigen
PVDF: Poly-vinylidene difluoride
YEPD: Yeast Extract-Peptone-Dextrose
