# Supplementary material for: The budding yeast protein Chl1p is required for delaying progression through G1/S phase after DNA damage
Source: Cell Div. 2021 Sep 8;16:4. doi: 10.1186/s13008-021-00072-x (PMC8424871; doi:10.1186/s13008-021-00072-x)

**Additional file 1 figure legend:**

**Additional file 1: Fig. S1. Chl1p follows the replication checkpoint pathway*.* A.** *G1/S-phase progression of single mutant and double mutant cells in the presence of MMS.* Mutant cell 699∆sgs1 (*sgs1*) and the double mutants, SL21 (*rad24sgs1*) and 699∆sgs1Dchl1 (*sgs1chl1*) were arrested at G1 by treating the log phase cells with alpha factor for 90 minutes at 30°C. 0.2% MMS was added in presence of the G1 block. All the cultures were kept shaking at 30°C. Aliquots were removed at various times of MMS exposure for FACS analysis. 0’ was collected just after adding 0.2% MMS to the cells with alpha factor (G1-blocked) followed by 10’, 20’ and 30’ of exposure to 0.2% MMS in presence of alpha factor (G1-blocked damaged cells). The histogram plots at each time point are overlayed in the figure by using overlay software (Guava- Incyte) to understand the progression of the cells through cell cycle. The exponential cells were collected just before addition of alpha factor to the growing cells of 0.2 OD_610nm_. Arrows indicate G1 and G2 DNA contents.


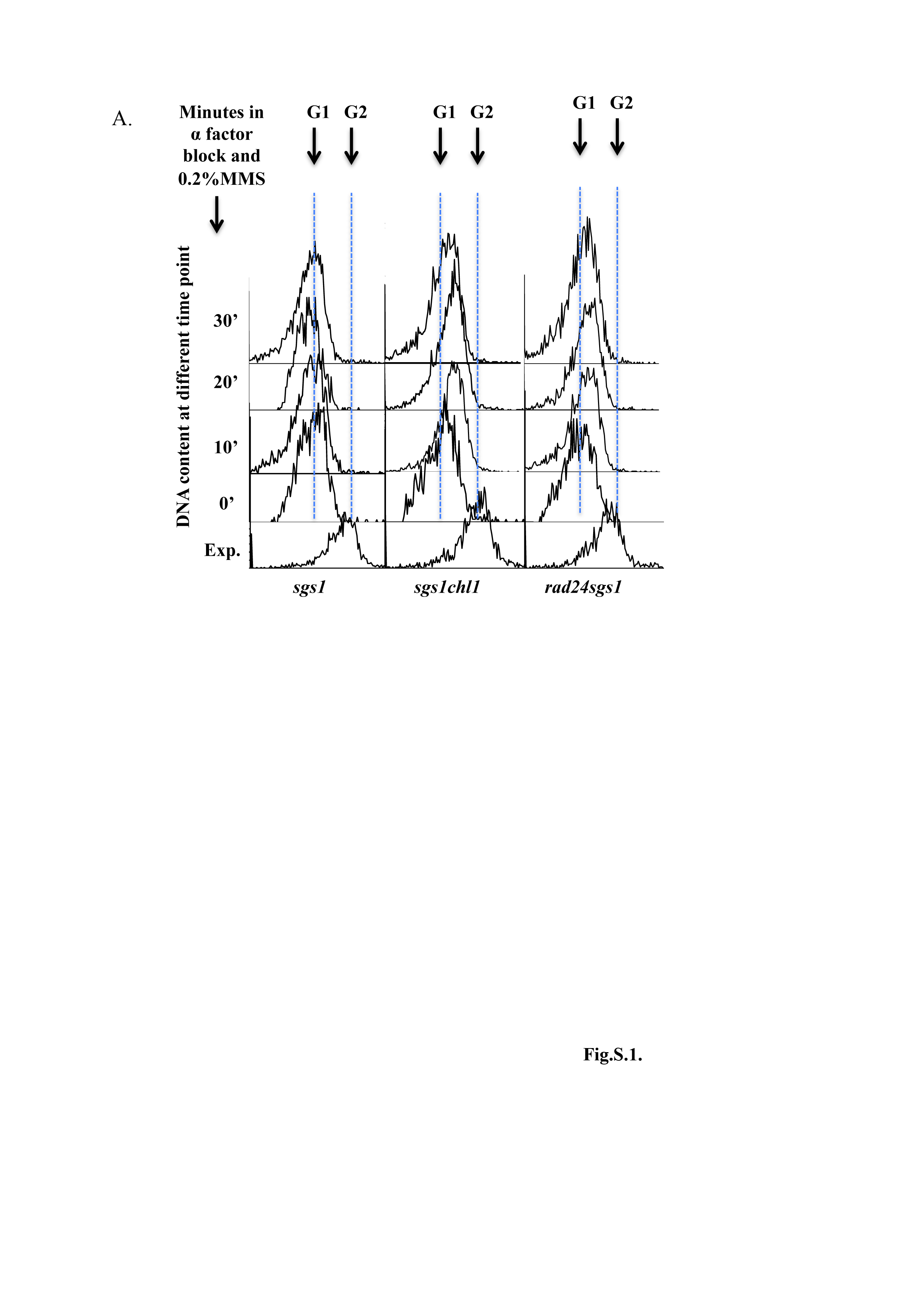

Supplement: Supplementary file 1 — Additional file 1: Fig. S1. Chl1p follows the replication checkpoint pathway. A G1/S-phase progression of single mutant and double mutant cells in the presence of MMS. Mutant cell 699∆sgs1 (sgs1) and the double mutants, SL21 (rad24sgs1) and 699∆sgs1Dchl1 (sgs1chl1) were arrested at G1 by treating the log phase cells with alpha factor for 90 min at 30 °C. 0.2% MMS was added in presence of the G1 block. All the cultures were kept shaking at 30 °C. Aliquots were removed at various times of MMS exposure for FACS analysis. 0’ was collected just after adding 0.2% MMS to the cells with alpha factor (G1-blocked) followed by 10’, 20’ and 30’ of exposure to 0.2% MMS in presence of alpha factor (G1-blocked damaged cells). The histogram plots at each time point are overlayed in the figure by using overlay software (Guava-Incyte) to understand the progression of the cells through cell cycle. The exponential cells were collected just before addition of alpha factor to the growing cells of 0.2 OD610nm. Arrows indicate G1 and G2 DNA contents. [file 13008_2021_72_MOESM1_ESM.docx]
